# Programmed cell death in hepatic fibrosis: current and perspectives

**DOI:** 10.1038/s41420-023-01749-8

**Published:** 2023-12-12

**Authors:** Ju-Lu Lu, Chuan-Xin Yu, Li-Jun Song

**Affiliations:** National Health Commission Key Laboratory of Parasitic Disease Control and Prevention, Jiangsu Provincial Key Laboratory on Parasite and Vector Control Technology, Jiangsu Provincial Medical Key Laboratory, Jiangsu Institute of Parasitic Diseases, 214064 Wuxi, Jiangsu People’s Republic of China

**Keywords:** Immune cell death, Target identification

## Abstract

The initiation, development and resolution of hepatic fibrosis are influenced by various cytokines, chemokines, damage-associated molecular patterns (DAMPs) and signaling pathways. A significant number of studies in recent years have indicated that the progression of hepatic fibrosis is closely linked to programmed cell death processes such as apoptosis, autophagy, pyroptosis, necroptosis, ferroptosis, cuproptosis, and PANoptosis. Inducement of hepatic stellate cells (HSCs) death or preventing death in other liver cells can delay or even reverse hepatic fibrosis. Nevertheless, the roles of programmed cell death in hepatic fibrosis have not been reviewed. Therefore, this review summarizes the characteristics of various of hepatic fibrosis and programmed cell death, focuses on the latest progress of programmed cell death in the promotion and regression of hepatic fibrosis, and highlights the different roles of the programmed cell death of HSCs and other liver cells in hepatic fibrosis. In the end, the possible therapeutic approaches targeting programmed cell death for treating hepatic fibrosis are discussed and prospected.

## Facts

- The programmed cell death is the potential target for treatment of hepatic fibrosis.
- The development of small molecule inhibitors for programmed cell death is helpful to slow or even reverse the progression of hepatic fibrosis.
- Programmed cell death occurs in different cell types and has different effects on hepatic fibrosis.

## Open questions

- Various types of programmed cell death crosstalk. How the crosstalk impact on hepatic fibrosis?
- How does drugs target HSCs without affecting healthy hepatocytes to reverse hepatic fibrosis?
- Immune cells in the liver are sensitive to programmed cell death. How the relationship contributes to hepatic fibrosis?
- The role of cuproptosis and PANoptosis, which are newly discovered forms of programmed cell death, in the development of hepatic fibrosis has not been reported.

## Introduction

Hepatic fibrosis is a frequently occurring pathological process in the progression of a number of chronic liver conditions and is characterized by long-term liver injury that activates quiescent hepatic stellate cells (HSCs), which transform into fibroblasts and continue to secrete large amounts of extracellular matrix (ECM), and excessive deposition in the extracellular interstitium [1]. Inhibiting HSCs activation or conversion to a nonactivated state can reverse hepatic fibrosis, which can be a therapeutic strategy [2]. Hepatic fibrosis can be the cause of cirrhosis or even cancer if not effectively treated. However, no clinically approved drugs have been reported to treat hepatic fibrosis [3], and the hepatic fibrosis mechanisms are intricate and not yet fully known [4]. Hence, it is crucial to elucidate its molecular mechanism and identify novel drug candidates. Programmed cell death is a fundamental component of organismal growth and development, and it substantially contributes to the host’s protection against pathogens, maintenance of homeostasis and participation in pathological processes. The different types of cell death seen in hepatic fibrosis propose that programmed cell death may be responsible in the formation and progression of hepatic fibrosis. Therefore, we review the latest advancement of programmed cell death in hepatic fibrosis, then point out the problem and its perspective, which may be helpful to provide new ideas to reverse the development of fibrosis.

## Hepatic fibrosis

Hepatic fibrosis is the result of repairing chronic damage, which is necessary in the promotion of chronic liver conditions towards cirrhosis [5]. Liver damage or hepatocyte death, triggered by alcohol, drugs, viral hepatitis, biliary stasis, parasite infections and nonalcoholic fatty damage, can be engulfed and activated by macrophages or hepatic stellate cells (HSCs), releasing profibrotic cytokines [6]. Immune cells can be triggered by damage associated molecular patterns (DAMPs) released by damaged hepatocytes, including high mobility group protein B1 (HMGB1), interleukin (IL)-33, adenosine triphosphate (ATP), etc. [7]. Neutrophils can contribute to HSCs activation by releasing reactive oxygen species (ROS) [8]. Liver sinusoidal endothelial cells (LSECs) can maintain the quiescent HSCs phenotype and prevent HSCs activation through their production of nitric oxide (NO) in the healthy liver [9]. CD4^+^ T cells are important drivers of the development of liver injury to fibrosis through the release of cytokines, and their different subtypes have different roles and mechanisms. Activated hepatic stellate cells, which transform into myofibroblasts, synthesis and secrete extracellular matrix (ECM), predominantly collagen types I (Col-I) and Col-III, which are excessively deposited in the damaged liver tissue, eventually leading to fibrosis [6]. We summarize the main characteristics of various types of hepatic fibrosis as follows.

### Hepatic fibrosis caused by alcohol, drugs or chemical toxicity

Alcohol is a leading contributor to hepatic fibrosis, cirrhosis and failure [10]. Ethanol can be metabolized into acetaldehyde by ethanol dehydrogenase in the liver. Excessive ethanol intake causes the retention of acetaldehyde in liver cells, which modifies protein and enzyme groups, leading to the irreversible formation of acetaldehyderatoms, hepatocellular misfunction and eventual cell death [11]. Alcohol intake is significantly and positively associated with fibrosis, and alcohol can increase the risk of fibrosis advancement in chronic liver diseases associated with hepatitis virus and drug damage [12].

In the liver, the accumulated drug metabolites can act as haptens to stimulate autoantibodies, leading to drug-induced liver injury (DILI) and then hepatic fibrosis [13]. Although the incidence of DILI is less prevalent than that of other causes, including alcohol, hepatitis, or steatosis, it can also result in severe complications.

Many chemicals, such as carbon tetrachloride (CCl_4_), thioacetamide, dimethylnitrosamine and diethylnitrosamine, accumulated in the liver, have also been shown to cause damage in the liver and to induce hepatic fibrosis. Due to their reproducibility and ease of use, these agents are often used in laboratory animal models, CCl_4_ being the most common [14].

### Hepatic fibrosis caused by viral hepatitis

Chronic infection with hepatitis B virus (HBV) and hepatitis C virus (HCV) of viral hepatitis has been identified as the leading cause of hepatic fibrosis over the past few decades. The persistence of the virus can lead to massive hepatocellular necrosis and inflammation, eventually evolving into hepatic fibrosis [7]. Hepatic fibrosis can be accelerated by overlapping infection with HBV and HCV [15]. With the development of antiviral treatments, the infection rate of viral hepatitis in adolescent has been significantly reduced, but the overall infection rate is still underestimated [16].

### Hepatic fibrosis caused by cholestasis

Cholestatic diseases are caused by genetic disorders (e.g., Alagille syndrome, Wilson disease and citrate malabsorption), mechanical damage to the bile duct (e.g., biliary fibrosis in pancreatopathy) or immune disorders (e.g., primary biliary cholangitis (PBC) and primary sclerosing cholangitis (PSC)), which then lead to damage or interruption of bile production and cause accumulation of toxic bile components, bile salts for example, resulting in cell death. If not treated in time, cholestasis can cause hepatic fibrosis and cirrhosis, eventually leading to liver failure and hepatoma. PBC and PSC, which are autoimmune cholestatic liver diseases, indicate the close connection between immune diseases, inflammatory responses and hepatic fibrosis [17].

### Hepatic fibrosis caused by parasite infection

Various parasites can cause hepatic fibrosis, such as schistosomes, *Clonorchis sinensis*, echinococcus and amoebae. Among them, schistosomes are the most widely studied [18]. Schistosomiasis hepatic fibrosis is characterized by egg granulomas and collagen deposition [19]. Eggs produced by adults schistosomes, depositing in the intestine and liver (*Schistosoma japonicum* and *Schistosoma mansoni*) or in urinary and reproductive tracts (*Schistosoma haematobium*). Soluble antigens are released by mature eggs to stimulate the immune response and differentiate CD4^+^ T cells into Th1 and Th2 cells. It was found that Th2 immune responses assume a dominant role in hepatic fibrosis caused by hepatosplenic schistosomiasis [20]. It has also been demonstrated that cytokines such as IL-13, transforming growth factor (TGF)-β1 and tumor necrosis factor (TNF)-α promote hepatic fibrosis, while cytokines such as interferon (IFN)-γ and IL-10 inhibit hepatic fibrosis [21]. In addition, DAMPs produced by hepatocyte necrosis, including IL-33 and HMGB1, can induce the Th2 immune response and stimulate the proliferation and activation of HSCs, thereby promoting hepatic fibrosis [22]. Besides, *Clonorchis sinensis* parasitizes in the host’s hepatobiliary duct. The mechanical stimulation and the excretion and secretion of the worm can lead to cholestasis of the hepatobiliary duct, inflammation of the bile duct, proliferation and thickening of the bile duct epithelium, and ultimately lead to hepatic fibrosis and hepatobiliary carcinoma [23].

### Hepatic fibrosis caused by nonalcoholic fatty damage

Nonalcoholic fatty liver disease (NAFLD) is the most common cause of liver disease in the developed world. As a collective term for a series of liver injuries and fibrosis, there are two known types of NAFLD: non-alcoholic fatty liver (NAFL) and non-alcoholic steatohepatitis (NASH). NAFL is typically benign, whereas NASH exhibits balloon-like hepatocellular damage and is a developmental stage of hepatic fibrosis and cirrhosis [24]. When hepatocytes are exposed to nonalcoholic fatty damage, immune cells release inflammatory factors such as TGF-β, TNF-α and IL-1, resulting in the activation of HSCs and ultimately lead to hepatic fibrosis [25].

## The role of programmed cell death in hepatic fibrosis

Cell death is a requirement condition for the normal growth and development of organisms. Depending on its controllability, it can be categorized into nonprogrammed and programmed cell death. Nonprogrammed cell death is the result of exposure of cells to harsh physical or chemical stimuli in the environment. Nonprogrammed cell death is an unregulated, passive process that cannot be blocked by inhibitors of cell signal transduction. It is characterized by ruptured cell membranes and swollen cells and organelles, but there is no chromatin condensation [26].

Programmed cell death is prevalent in developing organisms and important in maintaining homeostasis. When cells are provoked by the internal and external environment, they are regulated by genes and sequentially initiate protective suicide measures, which are regulated by various cell signaling molecules. Different modes of programmed cell death have been identified, as follows apoptosis, autophagy, pyroptosis, necroptosis, ferroptosis, cuproptosis and PANoptosis [27], and the characteristics of programmed cell death can be seen in Table 1.

**Table 1:** Comparison of types of programmed cell death.

	Apoptosis	Autophagy	Pyroptosis	Necroptosis	Ferroptosis	Cuproptosis	PANoptosis
Morphological characteristics	Reduced cell volume, broken nucleolus, blistered plasma membrane	Autolysosome formation	Cell swelling, cell membrane gap formation, cell rupture	Cytosolic effervescence, chromatin fixation, DNA cleavage	Smaller mitochondria, increased membrane density and reduced cristae	Mitochondrial wrinkling and mitochondrial membrane rupture	Cell swelling and rupture of the cytosol
Biochemical characteristics	Activation of DNA fragments and macromolecular synthesis	Increased lysosome activation	Caspase-1 activation dependent/noncaspase dependent, GSDMD cleavage and inflammatory factor release	Activation of RIP1, RIP3, and MLKL and a decrease in ATP levels	Decreased expression of GSH and GPX4, increased divalent iron and lipid peroxidation	Cu^2+^ binds to lipoacylated DLAT and induces heterodimerization of DLAT	PANoptosome assembly
Positive regulators	Bax Bak p53	LC-3 ATG4 ATG7	Caspase-1/11 GSDMD	RIP1 RIP3 MLKL	Ras P53 NOX CARS	FDX1 LIPT1 LIAS DLD	ZBP1 AIM2 RIP1 TAK1
Negative regulators	Bcl-2 Bcl-xL	–	GPX4 PKA	–	GPX4 SLCA711	–	–
Inflammation	No	No	Yes	Yes	Yes	Yes	Yes
Effector proteins	Caspase-3/6/7	LC3-II	GSDMD	MLKL	PLOOHs	Lipoylated proteins	Caspase-3/6/7 Caspase-1/4/5/11 MLKL
Inducer	FASL UNC5B DCC	Rapamycin Sodium valproate C2-ceramide EBSS	ZnO-NPs Ivermectin	TNFα Z-VAD-FMK DAMPs	Erastin RSL3 BSO SAS Sorafenib	Elesclomol	IRF1 TNF-α IFN-γ
Inhibitors	XIAP ILP-2 Z-VAD-FMK c-IAP-1 c-IAP-2	3-MA SAR-405 Spautin-1 Hydroxychloroquine Bafilomycin A1	Disulfiram	Nec-1 Necrosulfonamide	Deferoxamine Chicotic acid Ferrostatin-1 FSP1 GCH1	Tetrathiomolybdate	–
References	[28–43, 104]	[44–57]	[58–70, 104–106]	[71–78, 104–107]	[79–92, 108–110]	[93–98]	[99–102]

**Fig. 1:**
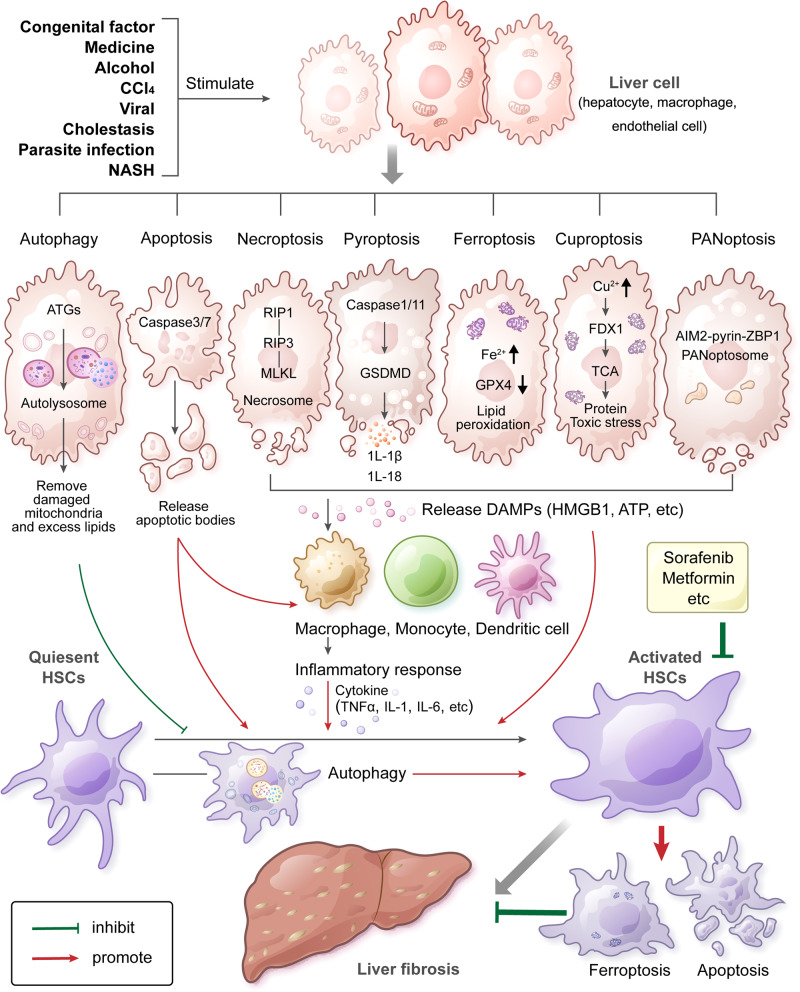
Overview of the role of programmed cell death in hepatic fibrosis. In response to congenital factors, medicine, alcohol, CCl_4_, viral, cholestasis, parasite infection, and NASH, liver cells including hepatocyte, macrophage, and endothelial cells, undergo autophagy, apoptosis, necroptosis, pyroptosis, ferroptosis, cuproptosis, or PANoptosis. Autophagy is regulated by ATGs and is mediated by the formation of autolysosomes, which remove damaged mitochondria and excess lipids, thereby inhibiting the activation of HSCs and promoting hepatic fibrosis. In apoptotic cells, activation of caspase-3 and caspase-7 leads to the formation of apoptotic bodies, which activate HSCs directly or by activating macrophages. In necroptotic cells, activation of RIP1-RIP3-MLKL signaling causes the formation of necrosomes, resulting in cell membrane rupture. Activated caspase-1 and caspase-11 cleave GSDMD proteins to perforate cell membranes and promote the secretion of the inflammatory factors IL-1β and IL-18, with the consequent development of pyroptosis. Apoptosis, pyroptosis, and necroptosis are collectively referred as PANoptosis. Upstream of PANoptosis, the key molecules AIM2, pyrin, and ZBP1 regulate the formation of PANoptosomes, ultimately leading to cell membrane rupture. The main characteristic of ferroptosis is lipid peroxidation, which is resulted from the excessive enrichment of iron-dependent ROS in cells and the weakened clearance of GPX4. Those changes lead to decreased mitochondrial size, increased membrane density and mitochondrial membrane rupture. Cuproptosis, regulated by FDX1, is caused by the direct interaction of copper ions with fatty acylated proteins involved in the TCA cycle of mitochondrial respiration. Such interactions lead to protein toxic stress and ultimately to mitochondrial membrane rupture. Necroptosis, pyroptosis, ferroptosis, cuproptosis and PANoptosis can release DAMPs, including HMGB1, ATP, and IL-1, which lead to the aggregation of macrophages, monocytes and dendritic cells, the secretion of inflammatory factors such as TNF-α, IL-6, and IL-1, and further expand the inflammatory response. Inflammation stimulates the activation of quiescent HSCs, which then promote hepatic fibrosis. Autophagy in quiescent HSCs includes lipid degradation, also called lipophagy, which in turn leads to lipid droplet mobilization and mitochondrial β-oxidation to provide energy for HSCs activation. Small-molecule compounds such as sorafenib and metformin can induce ferroptosis and apoptosis in activated HSCs, inhibiting hepatic fibrosis. In short, programmed cell death in different cell types has different effects on hepatic fibrosis. Promotion and inhibition of hepatic fibrosis are indicated by the red line and the green line, respectively. NASH nonalcoholic steatohepatitis, ATG autophagy-related gene, HSC hepatic stellate cell, GPX4 glutathione peroxidase 4, FDX1 ferredoxin 1, ROS reactive oxygen species, TCA tricarboxylic acid, TNF tumor necrosis factor, IL interleukin, DAMP damage-associated molecular pattern, ZBP1 Z-DNA binding protein 1, AIM2 absent in melanoma 2, RIP receptor interaction protein kinase, MLKL mixed-lineage kinase domain-like pseudokinase, GSDMD Gasdermin D, ATP adenosine triphosphate.

The liver, which is an important defense against various pathogenic microorganisms and their products, can activate programmed cell death when stimulated by DAMPs and pathogen-associated molecular patterns (PAMPs) in response to associated damage. The dead cells induce an inflammatory reaction to mediate natural immunity, leading to the activation, proliferation, differentiation of HSCs and the secretion of ECM through autocrine or paracrine signaling, resulting in matrix deposition and fibrosis [28]. HSCs can reverse hepatic fibrosis through cell death. Therefore, understanding the profibrotic effect of liver cell death and the antifibrotic effect of HSCs death is crucial for in-depth research on fibrosis mechanisms and the discovery of drug targets. The overview of the role of programmed cell death in hepatic fibrosis can be seen in Fig. 1.

### Apoptosis in hepatic fibrosis

Apoptosis, which was proposed by Kerr and colleagues in 1972, is an irreversible form of death characterized by a cascading reaction caused by the activation of cysteine aspartate-specific proteinase (caspase), which is widely present in the initiation and development of various diseases [29]. Apoptosis is mainly divided into three main pathways: extrinsic, mitochondrial and endoplasmic reticulum stress. The external pathway is mediated by the tumor necrosis factor (TNF) superfamily [30]. As a transmembrane protein, TNF can bind to its ligand on the cell membrane, attract Fas-associated death domain (FADD) proteins in the cytoplasm, form an apoptosis-inducing complex, activate caspase 8, and cause a downstream cascade reaction leading to apoptosis. The internal pathway is activated by the B-cell lymphoma-2 (Bcl-2) family via mitochondrial outer membrane permeabilisation (MOMP) [31]. The release of cytochrome C (cyt C) is crucial, as it binds with apoptosis-associated factor 1 (Apaf-1) to create a polymer in the presence of ATP. This polymer promotes caspase-9 to bind with it, resulting in the formation of apoptotic bodies [31]. Activated caspase-9 has the potential to activate other effector apoptotic proteases, such as caspase-3 and caspase-7, leading to apoptosis. In addition, when Ca^2+^ imbalance and misfolded proteins increase, the endoplasmic reticulum will be stressed, thus regulating Bcl-2 family proteins to initiate apoptosis [26]. Apoptotic cells can experience shrinkage and a decrease in volume, as well as nuclear concentration and rupture. However, the cell membrane is intact, and the resulting apoptotic bodies can be engulfed by adjacent macrophages without causing an inflammatory response [32]. In recent years, caspase-3 has been shown to induce apoptosis only after it enters the nucleus. Inhibiting its redistribution results in the loss of cell death sensitivity. Therefore, caspase-3 nuclear transport plays a crucial role in inducing apoptosis [33].

Increasing evidence suggests that apoptosis close association with hepatic fibrosis development. As early as 2001, Pianko and his colleagues confirmed that hepatocyte apoptosis was exacerbated in HCV patients combined ethanol intake with epidemiological analyses and demonstrated that HCV induced apoptosis through Fas/Fas ligand (FasL) interactions. Tan and his team induced hepatic fibrosis in mice by injecting CCl_4_, and they found expression upregulation of Fas, FasL, caspase-3, α-smooth muscle actin (SMA), Col-I and Col-IV, suggesting that hepatocyte apoptosis regulated by Fas/FasL was involved in in hepatic fibrosis [34]. p53-upregulated modulator of apoptosis (PUMA) is a target of Fas/FasL signaling and a key mediator of apoptosis. It is expressed at low or almost non-existent levels in normal cells and tissues [35], but can be rapidly upregulated in reaction to numerous stimuli [36]. PUMA has been shown to cause mitochondrial dysfunction and caspase activation via inhibiting other members of Bcl-2 family, including Bcl-2, Mcl-1 and Bcl-xL [36]. In a CCl_4_-induced hepatic fibrosis model, PUMA-KO knockout mice had fewer apoptotic cells and lower levels of fibrosis than PUMA-WT wild-type mice, indicating that PUMA-induced hepatocyte apoptosis promoted the formation of hepatic fibrosis [34]. Further research has demonstrated that apoptosis directly affects HSCs through the release of apoptotic bodies or by activating macrophages, leading to the activation of HSCs and hepatic fibrosis [37].

Notably, induction of HSCs apoptosis helps to clear activated HSCs and reverse hepatic fibrosis, which is currently the most reported effective treatment for hepatic fibrosis [38]. Zhou and colleagues used Fasudil to treat hepatic fibrosis of schistosomiasis, which can lead to apoptosis of HSCs and downregulate Col-I, Col-III, and TGF-β1 to reduce hepatic fibrosis caused by schistosomiasis [39]. M1 Bone-marrow-derived macrophages (BMDMs) could recruit and activate endogenous macrophages and natural killer (NK) cells, leading to apoptosis of HSCs and alleviating hepatic fibrosis [40]. A team found that CD69^+^CD103^-^CD8^+^ Trm was enriched when hepatic fibrosis subsides in NASH mouse, and confirmed that CD8^+^ Trm could attract HSCs and mediate apoptosis through Fas/FasL pathway, leading to the resolution of hepatic fibrosis [41]. γδ T cells were recruited into the liver by the cytokine receptor CCR6 during CCl_4_ chronic injury and promoted HSCs apoptosis through FasL, protecting the liver from excessive inflammation and fibrogenesis [42]. Recent studies have found that metformin can improve liver function and hepatic fibrosis induced by NASH by upregulating BIM, BAD and PUMA and downregulating Bcl-2 and Bcl-xL to induce apoptosis in HSCs [43], which provides a new approach for the management of hepatic fibrosis.

### Autophagy in hepatic fibrosis

Christian de Duve first discovered lysosomes in 1955 and defined autophagy in 1963 [44]. Autophagy is the process of degrading cell organelles and specific proteins into lysosomes, mediating cell stability and regeneration. Autophagy is regulated by autophagy-related genes (ATGs), and it is broadly divided into five processes [45]. After the cells are stimulated, the downstream serine/threonine protein kinase (ULK1/2) is activated, forming the ULK1 complex with ATG13 and ATG101, which activates the phosphatidylinositol 3-kinase (PIK3C3) complex to initiate autophagy. Next, the Atg5-Atg12-Atg16L complex forms, initiating the extension of autophagic precursors. Subsequently, microtubule-associated protein 1 light chain 3 (LC3) switches from soluble (LC3-I) to lipid-soluble (LC3-II) under the action of ATG4, which assists the maturation of autophagosomes. Then, in response to related genes, autophagosomes associate with lysosomes to produce autophagic lysosomes. Finally, the activity of sirolimus target protein (mTOR) is increased, which promotes the maturation and function of lysosomes, degrades their intracellular contents, and completes the autophagy process. ATGs are extremely important in the autophagy process. Earlier studies have proven that ATG1-10, 12–14, 16 and 18 perform crucial roles in the formation of autophagosomes and are the core ATG proteins of autophagy [46].

Recent studies have found that the levels of autophagic vacuoles and LC3-II in liver injury models are significantly increased. However, treating mouse and human HSCs with bafilomycin A1, an inhibitor of autophagy, can significantly reduce proliferation and the expression of activation markers [47]. In contrast, another study showed that autophagy could drive the activation of HSCs by regulating lipid droplets. When autophagy in HSCs was blocked with 3-methyladenine or specific siRNAs against ATG5 and ATG7, HSCs activation was reduced, and hepatic fibrosis was alleviated [48]. These results suggest a close relationship between autophagy and hepatic fibrosis. Autophagy mediates lipid degradation, which leads to lipophagy, in turn leads to lipid droplet mobilization and mitochondrial β-oxidation to provide energy for HSCs activation [48]. A recent study by Cao and his team showed that E7046, the specific prostaglandin E2 (PGE2) and its receptor EP4 antagonist, significantly suppressed M2 macrophage-mediated autophagy in HSCs and ameliorated hepatic fibrosis caused by NAFLD in mice, confirming that PGE2/EP4 enhanced autophagy in HSCs via the Erk pathway to cause fibrosis [49]. In addition, it has been shown that carvedilol inhibits HSCs autophagy by increasing lysosomal pH and reducing hepatic fibrosis [50]. Doxazosin also inhibited HSCs autophagy and reduced hepatic fibrosis through activation of the phosphatidylinositol 3-kinase (PI3K)/protein kinase B (Akt)/mammalian target of rapamycin (mTOR) pathway [51]. The excretory/secretion products of *Clonorchis sinensis* (CsESPs) can induce autophagy of HSCs, causing their activation and participating in the formation of hepatic fibrosis [52].

Autophagy in HSCs enhances hepatic fibrosis, whereas autophagy in hepatocytes and macrophages inhibits hepatic fibrosis. Ni and colleagues showed that in hepatocyte-specific ATG5-knockout mice, hepatic fibrosis was exacerbated. Further studies showed that the loss of autophagy through the p62-keap1-nuclear factor erythroid 2-related factor 2 (Nrf2) pathway leads to sustained Nrf2 activation, which increases oxidative stress and inflammatory responses in hepatocytes, leading to hepatic fibrosis [53]. The lack of Farnesoid X Receiver (FXR) in hepatocyte can lead to disturbance of bile acid homeostasis, inhibit autophagy and the release of inflammatory factors in the liver, thereby exacerbating liver cell damage and fibrosis caused by schistosomiasis [54]. In addition to hepatocyte autophagy, a team has found that specifically blocking macrophage autophagy can increase alcohol-induced liver damage and inflammation [55]. Macrophage autophagy can reduce the secretion of inflammatory factors and fibrogenic cytokines, thereby preventing hepatic fibrosis [56]. Hepatocyte and macrophage autophagy have been shown to prevent fibrosis. Autophagy in hepatocytes helps to maintain cellular homeostasis and reduce oxidative stress, damage due to lipid peroxidation, and apoptosis, thereby preventing the development of hepatic fibrosis [57]. Macrophage autophagy reduces the secretion of inflammatory and fibrogenic cytokines, thus preventing hepatic fibrosis [56]. In summary, the autophagy role in hepatic fibrosis is double-edged and depends on the type of cell in which it occurs.

### Pyroptosis in hepatic fibrosis

Pyroptosis is a mode of inflammatory programmed cell death that was first proposed by Brennan and colleagues in the year 2000. It is characterized by inflammasome formation, caspases and gasdermin D (GSDMD) activation and robust proinflammatory cytokines release, which can be divided into classical and nonclassical pathways [58]. The classic pathway involves PAMPs or DAMPs being recognized by pattern recognition receptors (PRRs) in the cytoplasm, which then assemble with apoptosis-related molecular proteins (ASCs) to form couplets, activating procaspase-1. Caspase-1 cleavage of the N-terminus of GSDMD leads to pores formation in the cell surface membrane, forming nonselective channels that promote the secretion of inflammatory mediators, in particular IL-1β and IL-18, exacerbating the inflammatory response [59]. The assembly of non-selective pores promotes the flow of water, leading to cell edema, membrane foaming with vesicular protrusions (called pyroptosomes), loss of plasma membrane integrity and ultimately cell membrane disruption. Following disruption of the cell membrane, HMGB1 and ATP are released, with subsequent pyroptosis [60]. In the non-classical signaling pathway, caspase-4 and caspase-5 in human or caspase-11 in mice is activated by lipopolysaccharide (LPS), which can directly induce pyroptosis or indirectly induce pyroptosis by cleaving GSDMD to cause membrane perforation [61]. The role of caspase 1 and GSDMD as key mediators of pyroptosis needs to be further investigated.

Pyroptosis contributes to hepatic fibrosis through the release of DAMPs and inflammasomes [62]. Pyroptosis and hepatic fibrosis were induced by activating NOD-like receptor family pyrin domain containing 3 (NLRP3) inflammasomes in primary hepatocytes from mice, and blocking caspase-1 and GSDMD inhibited pyroptosis and hepatic fibrosis [63]. In a cohort study, caspase-1 was found in the serum of NASH patients and positively correlated with the severity of disease. Moreover, research has shown that mice overexpressing NLRP3 spontaneously developed to hepatic fibrosis. Liu and colleagues found that liver expression of GSDMD, NLRP3, caspase-1 and IL-1β increased in *Schistosoma japonicum*-infected mice, leading to exacerbation of hepatic fibrosis. In contrast, the level of hepatic fibrosis decreased in NLRP3 knockout mice infected with *Schistosoma japonicum*, suggesting that pyroptosis is involved in the hepatic fibrosis of schistosomiasis [64]. These studies indicate that NLRP3 is important in the induction of pyroptosis and fibrosis [65]. Another study reported that the level of caspase-11 in the liver from normal NASH mice was upregulated, while the liver injury, fibrosis, and inflammation in caspase-11-knockout mice was significantly decreased, and the level of caspase-11, GSDMD, and IL-1β was also reduced [66]. These findings indicate that caspase-11 modulating hepatocyte pyroptosis accelerates the advancement of NASH-induced hepatic fibrosis. Daphne found that a large number of eggs were deposited in the liver sinuses, leading to cell death, inflammatory response and fibrosis progression in mice infected with *Schistosoma mansoni*. Further studies showed that necrotic cells of the liver in mice with schistosome infection recruited eosinophils and underwent pyroptosis to promote hepatic fibrosis progression [67]. Schisandrin B (Sch B) of Schisandra chinensis can alleviate hepatic fibrosis caused by *schistosome mansoni* though inhibiting pyroptosis and inflammatory response [68]. Han and colleagues showed that Sestrin 2 hampered fibrogenesis caused by cholestasis via inhibiting NLRP3 inflammasome-mediated pyroptosis [69]. Moreover, Sphingomyelin synthase 1 (SMS1) mediates pyroptosis via the diacylglycerol (DAG)-protein kinase Cδ (PKCδ)- the NLR family CARD domain-containing protein 4 (NLRC4) axis. SMS1 holds promise as a novel drug target for the treatment of hepatic fibrosis [70].

### Necroptosis in hepatic fibrosis

Necroptosis, a novel programmed cell death, was discovered by Vercammen and colleagues in 1998 that has a typical necrotic morphology but upstream molecular regulation similar to apoptosis [71]. Necroptosis induced by the death receptor superfamily is the best studied. TNF-α is the most important factor mediating necroptosis. TNF-α recruits downstream proteins to form complex I by attaching to TNFR1 on the cell membrane. Complex I regulates receptor interaction protein kinase (RIP) 1 activation of downstream signaling pathways based on different environments and stimuli, which leads to cell survival, apoptosis, or necroptosis [72]. When caspase-8 is knocked down or inhibited, RIP1 recruits RIP3, and they interact through their RIP homology interaction motif (RHIM) to form necroptosomes, cause RIP3 phosphorylation and recruit mixed-lineage kinase domain-like pseudokinase (MLKL) to form the IIb complex. MLKL is phosphorylated by phosphor (p)-RIP3 to form tetramers and is inserted into the cell membrane, causing cell membrane breakdown and leakage of inflammatory cytokines, leading to tissue injury. RIP1, RIP3, and MLKL, which are regulatory factors, play crucial role in necroptosis [73].

Increasing evidences suggest that necroptosis is an important initiator of inflammation and hepatic fibrosis. RIP3 was upregulated in liver and macrophages from humans and mice suffering from hepatic fibrosis, while in RIP3-deficient macrophages of mice, hepatic fibrosis caused by CCl_4_ or bile duct ligation (BDL) was reduced [74], indicating an inseparable relationship between necroptosis and fibrosis. Further research has shown that AAV8-mediated knockdown of MLKL, which is the ultimate effector of the necroptosis signal pathway, significantly attenuates CCl_4_-induced hepatic fibrosis, indicating that MLKL-modulated necroptosis plays an essential role in liver disease and fibrosis. This research suggests that hepatic fibrosis can be blocked by reducing necroptosis in liver cells [75]. Yan and his colleagues found that specific knockout of endothelial MLKL in the NASH mouse model of hepatic fibrosis inhibited the activation of TGF-β/Smad 2/3 signaling pathway and reduced the degree of fibrosis, suggesting that endothelial MLKL could be a promising molecular target for the treatment of NASH fibrosis [76]. Song and colleagues showed that RIP3 was highly expressed in hepatocytes and CD45^+^ cells in hepatic fibrosis induced by schistosome infection. RIP3 regulates inflammation and ROS production through the c-Jun N-Terminal Kinase (JNK)-pJUN/Early growth response factor 1 (Egr1) signaling pathway, and knockdown of RIP3 reduces inflammation and hepatic fibrosis caused by schistosome infection [77]. This finding suggests a role of RIP3-mediated necroptosis in the promotion of hepatic fibrosis during schistosomiasis. Theoretically, necroptosis can be blocked at multiple levels by inhibiting the activity or phosphorylation of RIP1, RIP3 or MLKL. Multiple inhibitors targeting these molecules have been developed and patented, including various RIP1 inhibitors (such as Nec-1 and Nec-2), RIP1 and RIP3 inhibitors (such as GSKʹ840, GSKʹ843 or GSKʹ872), and MLKL inhibitors (such as necrosulfonamide) [78], which have shown good inhibitory effects on necroptosis and hepatic fibrosis at the animal and cell levels.

### Ferroptosis in hepatic fibrosis

Ferroptosis, formally proposed by Dixon’s team in 2012, is an iron-dependent programmed cell death distinct to necrosis, apoptosis and autophagy. It is characterized by glutathione (GSH) consumption and a decline in glutathione peroxidase 4 (GPX4) activity [79]. At present, ferroptosis is mainly regulated in three ways: glutamate/cystine transporter system xc^−^, GPX4 and iron metabolism. First, inhibiting the glutamate/cystine transporter leads to the disorder of extracellular cystine and intracellular glutamate replacement, and a reduction in intracellular cystine absorption, resulting in reduced synthesis of the antioxidant GSH, excessive accumulation of toxic ROS and reduced cellular antioxidant capacity, leading to cell death [80]. Second, a reduction in GPX4 activity prevents the conversion of lipid peroxide bonds to hydroxyl groups and prevents the cell membrane from repairing oxidative damage, leading to ferroptosis [81]. Third, excessive intracellular levels of divalent iron ions can generate much ROS through the Fenton reaction, which accumulate in the cell to cause oxidative damage and greatly increase the possibility of ferroptosis [82]. In recent years, several signaling pathways have been shown to regulate ferroptosis, including STAT3/p53/solute carrier family 7 member 11 (SLC7A11), bromodomain-containing protein 7 (BRD7)-p53-solute carrier family 25 member 28 (SLC25A28), and p62-Keap1-Nrf2 [83], which has implications for targeted therapy [84].

Ferroptosis is a major contributor to hepatic fibrosis. Iron overload and lipid peroxidation can be seen during hepatic injury and hepatic fibrogenesis. It has been demonstrated that feeding a high-iron diet to hepatocyte-specific Trf-knockout (Trf-LKO) mice increased their sensitivity to hepatic fibrosis, and treatment Trf-LKO mice with ferrostatin-1, a ferroptosis inhibitor, was effective in reversing the hepatic fibrosis caused by an iron-rich diet or by injections of CCl_4_ [85]. Su and his colleagues found that hepatocyte-specific TAK1 deficiency led to an imbalance of iron ions in hepatocytes, resulting in ferroptosis, which led to oxidative stress, activation of the macrophage STING signaling pathway and exacerbation of hepatic fibrosis, all of which were markedly attenuated by ferratine-1 treatment, suggesting that targeting ferroptosis of hepatocytes could be useful in the treatment of liver injury and hepatic fibrosis [86]. Wu and colleagues showed that fibroblast growth factor 21 (FGF21), which is a novel inhibitor of ferroptosis, was overexpressed in mouse hepatocytes to inhibit ferroptosis and rescue liver damage and fibrosis caused by iron overload [87]. The inhibition of hepatocyte ferroptosis reduces iron overload, lipid peroxidation and inflammatory infiltration during liver injury, thereby reducing hepatic fibrosis [88].

Interestingly, the role of ferroptosis in hepatic fibrosis has two sides, and the induction of ferroptosis in HSCs inhibits the formation of hepatic fibrosis. Dihydroartemisinin (DHA), which is a therapeutic drug for malaria, has recently been shown to reduce hepatic fibrosis in mice by inducing ferroptosis in HSCs [89]. Moreover, sorafenib, a molecular targeted drug for the treatment of advanced hepatocellular carcinoma in clinic, has also been demonstrated to alleviate hepatic fibrosis induced by CCl_4_ by triggering ferroptosis of HSCs via the Hypoxia-inducible factor 1 alpha (HIF-1α)/SLC7A11 pathway [90]. In addition, Sui and colleagues proved that magnesium isoglycyrrhizinate attenuated hepatic fibrosis and HSCs activation though modulating the ferroptosis pathway [91]. The ferroptosis inducer erastin can also alleviate hepatic fibrosis by inducing ferroptosis of HSCs, but cannot alleviate hepatic fibrosis by inducing ferroptosis of sinusoidal endothelial cells and macrophages [92]. In summary, triggering ferroptosis in HSCs or inhibiting ferroptosis in hepatocytes can effectively alleviate hepatic fibrosis, which can be new treatment strategies for hepatic fibrosis.

### Cuproptosis in hepatic fibrosis

Cuproptosis, first identified and named by Tevetkov’s team in 2022, is a novel copper-dependent cell death [93]. Extracellular Cu^2+^ can form a complex with specific ion carriers such as Elesclomol (ES) or Disulfiram (DSF) to cross the cytosol and enter the mitochondria, where Cu^2+^ can be transformed to the more toxic Cu^1+^ under the critical regulation of ferredoxin 1 (FDX1). Univalent copper and divalent copper can attach to fatty acylated proteins in the citric acid cycle [94], further inducing the assembling of acylated proteins and leading to deletion of Fe-S proteins, which results in protein toxicity, stress and ultimately cuproptosis [95]. After cuproptosis, mitochondrial shrinkage and mitochondrial membrane rupture can be observed. As a key gene for cuproptosis, FDX1 is closely related to changes in mitochondrial enzymes and deserves further research [93].

There is currently no relevant research on cuproptosis and hepatic fibrosis, but recently, a team has used public databases to constructed prognostic models of cuproptosis pattern-related genes (CRGs) in HCC to reveal the indispensable role of cuproptosis in the treatment of innate immunity and antitumor effects [96]. Moreover, copper-depleting nanoparticles (CDNs) have been shown to deplete copper accumulation in mitochondria, thereby inducing apoptosis in triple-negative breast cancer cells and achieving therapeutic effects [97], which offers a new thinking for further understanding cuproptosis and cancer treatment. Tomlinson and colleagues also showed that steroid 5β-reductase (AKR1D1), an important molecule participated in the inactivation of endogenous glucocorticoid and synthesis of catalytic bile acid, was attenuated with the development of NAFLD and could influence disease progression by regulating cuproptosis [98]. It is suggested that cuproptosis is involved in various inflammatory and tumor diseases and is likely to become a new target for the management of hepatic fibrosis.

### PANoptosis in hepatic fibrosis

Malireddi proposed the concept of PANoptosis in 2016 [99]. PANoptosis has the key characteristics of apoptosis, pyroptosis and necroptosis. At present, it is clear that Z-DNA binding protein 1 (ZBP1), absent in melanoma 2 (AIM2) and pyrin are upstream molecules that can facilitate the assembly of PANoptosome, which provide a molecular support for the binding of related proteins [100]. During viral infection, ZBP1 uses its zα1 domain to sense DNA and RNA viruses, while the RHIM domain can bind to homologous RIP3. The ZBP1-RIP3 complex is an activator of caspase-8-dependent apoptosis and MLKL-mediated necroptosis. In addition, ZBP1 modulates NLRP3 inflammasome activation via RIP3-caspase-8 mediating pyroptosis [101]. Specific blockade of ZBP1 vesicle assembly can completely inhibit the initiation of PANoptosis, which cannot be achieved by inhibiting the programmed cell death pathway alone [102]. In addition, knockdown of transforming growth factor-β-activated kinase 1 (TAK1) activity caused pyroptosis, apoptosis and necroptosis [103]. Recent studies have shown that adenosine deaminase of RNA1 (ADAR1), which is an RNA editor, can inhibit ZBP1-mediated PANoptosis [102].

Many researches have demonstrated that PANoptosis is associated with many diseases, such as autoimmune diseases, neurodegenerative diseases, cancer, microbial infections and metabolic diseases [101]. Recent studies have shown that ADAR1, which is an RNA editor, inhibits ZBP1-mediated PANoptosis, thereby promoting tumorigenesis [102]. Lee and colleagues also showed that during *Herpes simplex virus* (HSV1) and *F. novicida* infection, AIM2, Pyrin and ZBP1 form a multiprotein complex called the AIM2-PANtosome by interacting with ASCs to mediate PANoptosis against microbial infections [100]. However, PANoptosis has not been reported to correlate with hepatic fibrosis, and so an in-depth understanding of PANoptosis and its associated regulatory factors is critical for the treatment of hepatic fibrosis.

## Discussion

Recent findings have shown that programmed cell death, which is a key immunoregulatory factor, plays a significant role in the initiation, progression and resolution of hepatic fibrosis, suggesting that programmed cell death is the potential target for the treatment of hepatic fibrosis. The development of small molecule inhibitors for programmed cell death is helpful to slow or even reverse its progression. However, since various phenotypes have been observed at the animal or genetic level by knocking out or overexpressing a gene, the role and molecular mechanisms in humans are still unclear. Therefore, human samples of hepatic fibrosis should be used to verify the role of programmed cell death in hepatic fibrosis. And the therapeutic effect of small molecule inhibitors on hepatic fibrosis requires be tested in clinical trials in future.

In recent years, an increasing number of studies have shown that there are extensive interactions between various seemingly independent programmed cell death pathways. Caspase-8 serves as a molecular switch that determines whether cells are heading toward survival or apoptosis, pyroptosis, or necroptosis [104]. Caspase-8-deficient mice were shown to undergo embryonic death, while simultaneous knockout of RIP3 and MLKL resulted in survival, suggesting that caspase-8 could inhibit necroptosis mediated by RIP3 and MLKL [105]. Interestingly, caspase-8, which is a protein molecular scaffold, induced severe pyroptosis in the intestine when knocked out, suggesting that it is essential for preventing pyroptosis [104]. Caspase-3 and caspase-7 can also promote the transformation of cells from apoptosis to pyroptosis by cleaving GSDMD [106]. Besides, autophagy and apoptosis crosstalk via Beclin 1/Bcl-2/Bcl-xL structural interaction, while autophagy could either promote, inhibit or unrelated to necroptosis [107]. RIP3 of necroptosis increased ROS production and lipid peroxidation contribute to the occurrence of ferroptosis [108]. Recent studies have found that ferroptosis is an autophagy dependent programmed cell death, which was regulated by autophagy through degrading ferritin, the major iron storage protein complex, to increase iron levels to promote ferroptosis [109]. Ferroptosis is also closely related to apoptosis and pyroptosis, but the mechanism still needs further exploration [110]. The relationships between various types of programmed cell death are complex. Therefore, the upcoming challenge will be to understand their interaction mechanisms and relationships with the diseases.

In addition, programmed cell death occurs in different cell types and has different effects on hepatic fibrosis. Apoptosis and ferroptosis occur in hepatocytes and promote the formation of fibrosis, whereas in HSCs they inhibit fibrogenesis. Autophagy, on contrary, occurs in hepatocytes inhibiting inflammatory and protects the liver from fibrosis, whereas in HSCs autophagy leads to lipophagy and exacerbates fibrosis. Therefore, targeting HSCs to induce cell apoptosis or ferroptosis, or inhibiting autophagy is helpful for reversing fibrosis, which is currently an important approach for treating hepatic fibrosis. However, optimizing drugs to target HSCs without affecting healthy hepatocytes remains a challenge.

Immune cells in the liver, such as macrophages, T cells, B cells, and eosinophils, may be implicated in various modes of programmed cell death and have an impact on hepatic fibrosis. For example, macrophages or eosinophils involves in pyroptosis and necroptosis, promoting hepatic fibrosis. At the same time, immune cells are sensitive to programmed cell death, which plays a key role in the polarization of macrophages and the differentiation of CD4^+^ T and B cells [111, 112], modulating natural and adaptive immunity. However, the relationship contributes to hepatic fibrosis is poorly understood and warrants further study.

In summary, hepatocyte, macrophage or endothelial cell undergoes apoptosis, necroptosis, pyroptosis or ferroptosis, promoting the formation of hepatic fibrosis, which would be drug target for the treatment of hepatic fibrosis. It was reported that fibrosis can be reversed by targeting HSCs to induce cell apoptosis or ferroptosis, or by inhibiting autophagy. However, the role of cuproptosis and PANoptosis, which are newly discovered forms of programmed cell death, in the formation of hepatic fibrosis has not been reported, may provide us with ideas and directions for finding novel drug targets to treat hepatic fibrosis.
